# Anatomical study of the location of the antilingula, lingula, and mandibular foramen for vertical ramus osteotomy

**DOI:** 10.1186/s40902-018-0155-3

**Published:** 2018-07-25

**Authors:** Jin Hoo Park, Hwi-Dong Jung, Hyung Jun Kim, Young-Soo Jung

**Affiliations:** Department of Oral and Maxillofacial Surgery, Yonsei University College of Dentistry, 50-1 Yonsei-ro, Seodaemun-gu, Seodaemoon-Gu, Seoul, 120-752 South Korea

**Keywords:** Antilingula, Lingula, Mandibular foramen, Vertical ramus osteotomy

## Abstract

**Background:**

The purpose of this study was to identify the location of the antilingula, lingula, and mandibular foramen in Korean cadavers and to promote safe and accurate surgery without damage to the inferior alveolar neurovascular bundle (IANB) when performing a vertical ramus osteotomy (VRO).

**Methods:**

This study was conducted on the dried mandibles of 20 adult cadavers. Digital calipers were used to measure the distances from the anatomical reference points (antilingula, lingula, and mandibular foramen).

**Result:**

The antilingula was located at the anterior 44% and superior 31% in the ramus. The lingula was located at the anterior 55% and superior 30% in the ramus. The mandibular foramen was located at the anterior 58% and superior 46% in the ramus. Regarding the positional relationship with the antilingula, the lingula was located 0.54 mm superior and 4.19 mm posterior, and the mandibular foramen was located 6.95 mm inferior and 4.98 mm posterior. The results suggested that in order to prevent damage to the IANB, osteotomy should be performed in the posterior region of ramus at least 29% of the total horizontal length of the ramus.

**Conclusion:**

Using only the antilingula as a reference point is not guaranteed to IANB injury. However, it is still important as a helpful reference point for the surgeon in the surgical field.

## Background

Vertical ramus osteotomy (VRO) is widely used for the surgical treatment of mandibular deformity [1]. VRO is an advantageous technique in that it is less likely to damage the inferior alveolar neurovascular bundle (IANB), but if this structure is damaged, it may lead to amputation of the IANB. However, there is also a disadvantage in that osteotomy cannot be performed by directly observing the location of the IANB from the medial side of ramus [2]. For the above reasons, the anatomical location of the IANB was the most important consideration for the surgeon in performing VRO, and long-term studies have been conducted to determine anatomical reference points to prevent damage to the IANB. Many previous studies have identified the mandibular foramen, through which the IANB passes into the mandible, and the protruding anatomical structure that predicts the position of the lingula in front of the mandibular foramen, on the lateral side of the ramus; Yates named it the antilingula [3]. Several studies have since been conducted on the antilingula, lingula, and mandibular foramen [3–13].

Mandibular prognathism is a common maxillofacial deformity especially in East Asian populations, and treatment with VRO is effective in these cases [14]. However, no studies have been reported on direct measurement of such anatomical reference points in the mandibles of East Asian patients. This study attempted to locate the antilingula, lingula, and mandibular foramen by direct measurement of the East Asian mandible in order to reduce the risk of IANB injury, which may occur when performing VRO. Therefore, this study was to promote safe and accurate VRO.

## Methods

**Fig. 1:**
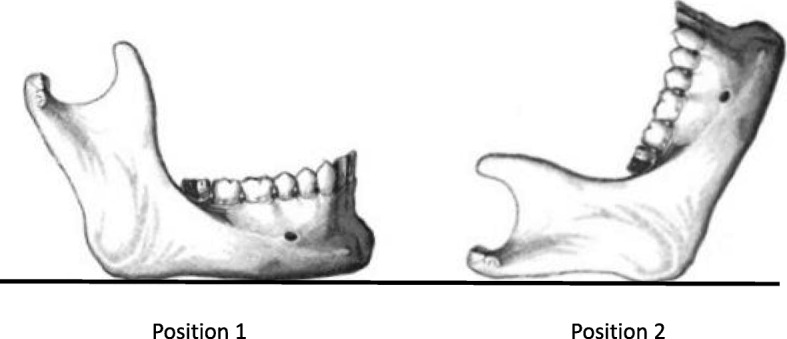
The measurement reference position. Position 1: the position wherein the lower margin of the mandible was placed on the platform. Position 2: the position wherein the posterior point of the condyle and the posterior point of the mandibular were placed on the platform

**Fig. 2:**
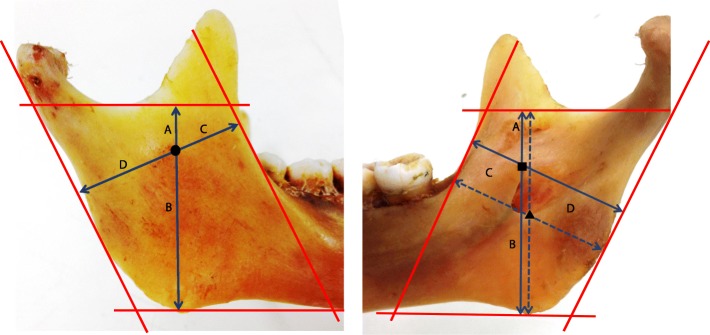
Antilingula (circle), lingula (square), and mandibular foramen (triangle) measurement. Antilingula: the most prominent point on the lateral surface of the mandibular ramus. Lingula: the most superior point of the lingual. Mandibular foramen: the lowest point of the entrance of the IANB into the mandible

This study was conducted on the dried mandibles of 20 Korean adult cadavers with age and sex unknown and < 4 missing teeth. All measurements were performed using a digital caliper fixed perpendicular to the platform, and the distances were measured from an anatomical measurement reference point up to 0.01 mm. For accuracy, each measurement was performed by a measurer who majored in oral maxillofacial surgery. The measurement reference points were “antilingula,” “lingula,” and “mandibular foramen.” Antilingula was the most prominent point on the lateral surface of the mandibular ramus and was confirmed by visual and tactile measurements. Lingula was the most superior point of the lingula, and mandibular foramen was the lowest point of the entrance of the IANB into the mandible. The measurement reference position was set to position 1 and position 2. Position 1 was the position wherein the lower margin of the mandible was placed on the platform, and position 2 was the position wherein the posterior point of the condyle and the posterior point of the mandibular were placed on the platform (Fig. 1). Measurements were made to determine distances A, B, C, and D. A was the distance between the line contacting the most concave point of the sigmoid notch and the anatomical measurement reference point parallel to the platform at position 1. B was the distance between the platform at position 1 and the anatomical measurement reference point. C was the distance between the line contacting the most concave point in the anterior of the ramus and the anatomical measurement reference point parallel to the platform at position 2. D was the distance between the line contacting the most concave point of the posterior of the ramus and the anatomical measurement reference point parallel to the platform at position 2 (Fig. 2). The mean and standard deviation of the distances (A, B, C, D), and the mean, standard deviation, maximum value, and minimum value of the distances between the antilingula and the other two anatomical measurement reference points were obtained. Pearson correlation coefficients were used to examine the correlations. The position of each anatomical reference point in the ramus was calculated as a ratio of the total length, and the mean, standard deviation, and maximum and minimum values were obtained. Statistical analysis was performed using Statistical Package for Social Sciences for Windows (version 22.0, SPSS, Chicago, IL, USA).

## Results

The antilingula was located an average of 14.61 mm (SD = 3.74) inferior from the sigmoid notch and 14.71 mm (SD = 1.63) posterior from the anterior border of the ramus. The lingula was located an average of 14.06 mm (SD = 3.05) inferior from the sigmoid notch and 18.89 mm (SD = 1.91) posterior from the anterior border of the ramus. The mandibular foramen was located an average of 21.56 mm (SD = 2.31) inferior from the sigmoid notch and 19.69 mm (SD = 2.22) posterior from the anterior border of the ramus (Table 1).

**Table 1:** Mean value (*M*) and standard deviation (SD) for each anatomic point

		Measurement			
Anatomic point		A	B	C	D
Antilingula	Mean (mm)	14.61	32.12	14.71	18.59
	SD	3.74	3.38	1.63	1.82
Lingula	Mean (mm)	14.06	32.36	18.89	18.89
	SD	3.05	3.49	1.91	1.91
Mandibular foramen	Mean (mm)	21.56	25.18	19.69	14.41
	SD	2.31	3.59	2.22	2.32

From the antilingula, the lingula was located 0.54 mm (SD = 2.87) superior from the sigmoid notch and 4.19 mm (SD = 2.25) posterior from the anterior border of the ramus. The maximum distances to the superior, inferior, anterior, and posterior sides were 6.24, 4.88, 0.12, and 9.14 mm, respectively. From the antilingula, the mandibular foramen was located 6.95 mm (SD = 3.11) inferior from the sigmoid notch and 4.98 mm (SD = 2.38) posterior from the anterior border of the ramus. The minimum and maximum distances to the inferior side were 1.04 and 12.86 mm, respectively, and the minimum and maximum distances to the posterior side were 0.16 and 12.86 mm, respectively (Table 2). Statistically, the antilingula and lingula (*r* = 0.659, *p* < 0.01) and the antilingula and mandibular foramen (*r* = 0.659, *p* < 0.01) exhibited moderate vertical correlations. However, the horizontal correlations between the antilingula and lingula (*r* = 0.202, *p* = 0.211), and the antilingula and mandibular foramen (*r* = 0.262, *p* = 0.102) were weak and statistically insignificant (Table 3).

**Table 2:** Mean value (*M*), standard deviation (SD), maximum (Max), and minimum (Min) value for difference between antilingula and other point

		Measurement	
Anatomic point		A	C
Antilingula and lingula	Mean (mm)	+ 0.54	+ 4.19
	SD	2.87	2.25
	Max./Min	+ 6.24/− 4.88	+ 0.12/− 9.14
Antilingula and mandibular foramen	Mean (mm)	− 6.95	+ 4.98
	SD	3.11	2.38
	Max./Min	−1.04/− 12.86	− 0.16/− 9.06

At “A” difference measurement, positive value means that antilingula inferior to other points and negative value means that antilingula superior to other points. At “C” difference measurement, positive value means that antilingula anterior to other points and negative value means that antilingula posterior to other points)

**Table 3:** Correlation among the measurements between the antilingula, lingula, and mandibular foramen

*r*	Lingula	Mandibular foramen		Lingula	Mandibular foramen
Antilingula (vertical)	0.659**	0.559**	Antilingula (horizontal)	0.202	0.262

Pearson correlation analysis: **p < 0.01

**Fig. 3:**
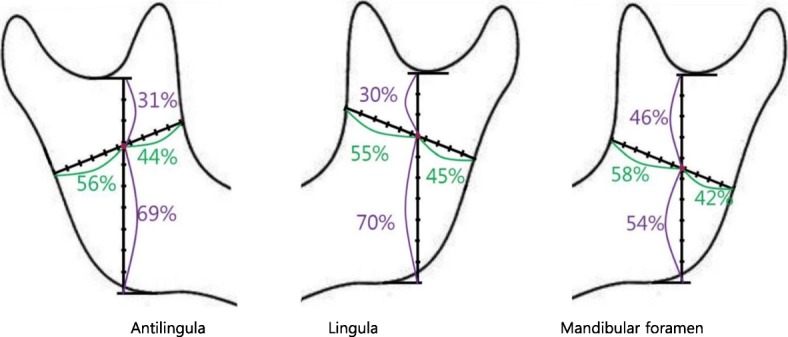
Average position of anatomic point on ramus. Minimum value from border of ramus. Anterior-***A***, posterior-***P***, superior-***S***, and inferior-***I***. Antilingula ***A***: 36%, ***P***: 48%, ***S***: 20%, ***I***: 56%. Lingula ***A***: 46%, ***P***: 38%, ***S***: 23%, ***I***: 51%. Mandibular foramen ***A***: 29%, ***P***: 47%, ***S***: 37%, ***I***: 47%

With regard to the ratio of each measurement reference point to the border of the ramus, the antilingula was 56% (SD = 4%) horizontal from the posterior border of ramus, and 31% (SD = 6%) vertical from the sigmoid notch. The lingula was 45% (SD = 5%) horizontal from the posterior border of the ramus, and 30% (SD = 5%) vertical from the sigmoid notch. The mandibular foramen was 42% (SD = 6%) horizontal from the posterior border of the ramus, and 46% (SD = 4%) vertical from the sigmoid notch. The lowest values of the anteroposterior and superoinferior ratios of the antilingula were 36 and 48%, and 20 and 56%, respectively. The lowest values of anteroposterior and superoinferior ratios of the lingula were 46 and 38%, and 23 and 51%, respectively. The lowest values of the anteroposterior and superoinferior ratios of the mandibular foramen were 29 and 47%, and 37 and 47%, respectively (Fig. 3).

## Discussion

Damage to the IANB during orthognathic surgery on the mandibular ramus is a major complication that can be avoided. To prevent this complication, there have been several attempts to develop novel surgical techniques to avoid damage to the IANB during osteotomy of the lateral side of the ramus, such as VRO, inverted L-osteotomy, and C-shaped osteotomy. In order to identify the theoretical basis of these surgical techniques, there have been many studies to determine the anatomical location of the IANB in the lateral side of ramus [3–6, 8–12].

The antilingula is an elevated part of the lateral side of the ramus that was previously described as a prominence [1], bump [15], or tubercle [16]. Yates et al. [3] referred to this structure as the antilingula and were the first to report a relationship with the mandibular foramen. In subsequent years, research on the antilingula was conducted as an anatomical measurement reference point for mandibular surgery. In a study by Yates et al. [3] using 70 dry mandibles, the antilingula was found in 44%, indefinitely found in 41%, and could not be found in 15%. Yates claimed that the antilingula was a highly variable anatomical landmark, but that the posterior 5~ 10 mm of the antilingula was a statistically safe area. Pogrel et al. [6] found the antilingula in all cases in a study of mandibles in 20 cadavers; in most cases, the lingula was present in the posteroinferior region of the antilingula. Aziz et al. [8] found the antilingula in all cases in a study of mandibles in 18 cadavers. The lingula was present in the anterior, posterior, superior, and inferior regions of the antilingula, but there was “no risk of damaging the neurovascular bundle” during osteotomy in the posterior 5 mm of the antilingula.

Recently, studies investigating Asian populations have also been introduced. In a study by Apinhasmit et al. [9] using 92 dry mandibles, the antilingula was found in 80.4% of the patients, and it was confirmed that the antilingula was primarily present in the anterior-inferior region of the lingula. In a study by Hosapatna et al. [12] using 50 South Indian dry mandibles, it was confirmed that the mandibular foramen existed in the posterosuperior region of the antilingula, in contrast to the findings of previous studies. In the present study, the lingula was present in posterosuperior region of the antilingula. Although the difference in the superior side was 0.54 mm, which was not significant, this was slightly different from previous studies. On the other hand, the mandibular foramen was found to be present in the posteroinferior region of the antilingula. This is a similar pattern with most existing studies.

The East Asian populations have more cases of mandibular prognathism than other races, and VRO can be useful in this case [14]. However, the anatomical studies of the mandibular ramus have been conducted mainly on Caucasian populations, and have been carried out in Southeast Asian, and Indian patients. In contrast, cases of direct measurement of the mandible in East Asian populations are not common. In this respect, the present study is meaningful.

As much as the interest in Antilingula, a controversy was raised. In a study by Reitzik et al. [4], in addition to antilingula, anatomical points termed the “midpoint of the waist of the ascending ramus” (MW) and the “midpoint of a line joining the coronoid process to the gonion” (MCG) were identified. The study reported that the MW was the most useful among the three anatomical points. Martone et al. [5] insisted that no antilingula was present and that the MW was the surgical reference point. Park et al. [11] used three-dimensional CT to study 25 patients with normal class 1 occlusion, 50 patients with mandibular prognathism, and 50 patients with mandibular retrognathism. The antilingula was clinically identifiable in 46.7, 44.4, and 45.3% of cases, and the MW was reported to be an excellent intraoperative reference point. Hogan and Ellis [17] reported that the antilingula is not an anatomical marker associated with the mandibular foramen and is not appropriate as a surgical guide for osteotomy because it is a musculotendinous apparatus. In a study by Monnazzi et al. [10] using 44 dry mandibles, antilingula was not recommended for use as a VRO landmark. In the present study, we found the antilingula in all subjects, but the use of antilingula alone as an anatomical reference point is not believed to prevent damage to the IANB.

There were several considerations when defining the anatomical measurement points in this study. In some studies, the antilingula was not observed, and it was thought that there was difficulty in setting the antilingula [3, 5, 6, 9, 12]. However, our study demonstrated that the antilingula was the most prominent part of the lateral side of the ramus, which was found by both visual and palpation methods, and was observed on both sides of the mandible (40 sites) in 20 cadavers. In the setting of the mandibular foramen, in order to safely preserve the IANB, the measurement point must be set behind the most posterior border of the mandibular foramen. However, the posterior border of the mandibular foramen is not clear. In this study, the accuracy and consistency of the measurements are maintained, as a relatively objective land mark, which is the most inferior point of mandibular foramen. The lingula was relatively clear, and there was no difficulty in setting up the point lingula.

Recently, the development of imaging technology such as CT has aided in confirming and measuring the course of the IANB in the preoperative plan [11, 18, 19]. However, in order for the surgeon to perform exactly the planned operation, it is necessary to know the accurate structure of the mandible to lower the risk of surgery, and information on the anatomical structures that can be directly observed in the surgical field are needed. Because the mandibular foramen and lingula are difficult to visually identify when performing VRO, the structure must be recognized by the surgeon. The antilingula is the most prominent part of the lateral side of the ramus and is easy to observe, even if this region does not define the exact position of the mandibular foramen, it can be highly useful as a reference point for the entire ramus.

## Conclusions

According to the results of this study, the surgical safe region, which we propose for safe and accurate surgery in order to prevent damage to the IANB during VRO, is 29% posterior region of the total horizontal length of the ramus, 37% superior region of total height of ramus from sigmoid notch to inferior border. Numerically, this region is posterior region more than 9.02 mm from the posterior border of the ramus and superior region more than 17.86 mm from the sigmoid notch.

The current study used mandibles from only 20 cadavers; therefore, further studies will be needed in the future. Additionally, due to the different anatomical characteristics of each individual, it is important to identify the anatomy of the patient via preoperative imaging to avoid damage during surgery.

Furthermore, when the antilingula alone is used as a reference point, prevention of damage to the IANB cannot be guaranteed. However, the antilingula is still important as a reference point for the surgeon in the surgical field. By locating the antilingula in the ramus, the surgeon may be able to approximate the overall anatomy of the ramus during surgery.

## Abbreviations

IANB: Inferior alveolar neurovascular bundle
VRO: Vertical ramus osteotomy
